# Vitamin D Status Among Infants Aged 1–5 Months in Northwestern Romania: A Single-Center Study and Implications for Infant Care and Management

**DOI:** 10.3390/nu18213471

**Published:** 2026-10-22

**Authors:** Ioana Badiu Tișa, Anamaria Cozma-Petruț, Elisabeta Ioana Hirișcău, Sorana D. Bolboacă

**Affiliations:** 1 Department of Nursing, Faculty of Nursing and Health Sciences, “Iuliu Hațieganu” University of Medicine and Pharmacy, 2-4 Câmpeni Street, 400217 Cluj-Napoca, Romania; ioana.badiu@umfcluj.ro (I.B.T.); ihiriscau@umfcluj.ro (E.I.H.); 2 Department of Bromatology, Hygiene, Nutrition, Faculty of Pharmacy, “Iuliu Hațieganu” University of Medicine and Pharmacy, 6 Pasteur Street, 400349 Cluj-Napoca, Romania; 3 Department of Medical Informatics and Biostatistics, Faculty of Medicine, “Iuliu Hațieganu” University of Medicine and Pharmacy Cluj-Napoca, 6 Louis Pasteur Street, 400349 Cluj-Napoca, Romania; sbolboaca@umfcluj.ro

**Keywords:** vitamin D status, 25-hydroxyvitamin D, vitamin D deficiency, hypervitaminosis D, infancy, vitamin D supplementation, breastfeeding, formula feeding, seasonal variation, Romania

## Abstract

**Background/Objectives:** Vitamin D is essential for calcium and phosphorus homeostasis and bone mineralization in infancy; serum 25-hydroxyvitamin D [25(OH)D] is its main biomarker. In Romania, data on vitamin D status among infants younger than six months remain limited, despite evidence of suboptimal status in other Romanian pediatric and perinatal populations. This study aimed to assess vitamin D status in infants aged 1–5 months in northwestern Romania and its associations with supplementation dose, feeding type, and season of blood sampling. **Methods:** This single-center observational study included infants aged 1–5 months. Serum 25(OH)D was interpreted as deficient (<12 ng/mL), insufficient (12–<20 ng/mL), sufficient (20–100 ng/mL), or potentially excessive (>100 ng/mL). Statistical analyses used parametric or non-parametric methods as appropriate to data distribution. **Results:** The cohort included 124 girls (56.6%) and 95 boys (43.4%; median age 4 [3–5] months). Median serum 25(OH)D was 61.24 [49.53–75.32] ng/mL; 94.5% of infants had sufficient concentrations, deficiency and insufficiency each occurred in 0.9%, and 3.7% had potentially excessive concentrations. Serum 25(OH)D increased progressively with reported supplementation dose (*p* < 0.0001) and differed by feeding type (*p* = 0.0279), with breastfed infants having higher concentrations than formula-fed infants. No significant seasonal difference was observed (*p* = 0.4218). **Conclusions:** In this hospital-based cohort, most infants had sufficient 25(OH)D concentrations, in a context of near-universal supplementation. Higher reported doses were associated with higher 25(OH)D concentrations, and concentrations >100 ng/mL occurred almost exclusively in infants receiving 2000 IU/day. Without data on calcium metabolism, these concentrations do not indicate vitamin D toxicity, but they support adherence to recommended prophylactic doses.

## 1. Introduction

Vitamin D is a secosteroid hormone with an essential role in calcium and phosphorus homeostasis, thereby supporting normal bone mineralization, skeletal growth, and development [1,2,3,4,5]. Its biologically active metabolite, 1,25-dihydroxyvitamin D [1,25(OH)_2_D], exerts its effects through the vitamin D receptor, which is expressed in numerous tissues and cell types [6]. Beyond its established role in bone metabolism, vitamin D is increasingly recognized as a modulator of immune function and inflammatory processes. Nevertheless, the clinical relevance of these extra-skeletal effects in children remains incompletely understood [7,8].

Vitamin D deficiency is an important global public health concern [9,10]. A pooled analysis of 308 population-based studies involving approximately 7.95 million participants from 81 countries reported serum 25(OH)D concentrations below 30 nmol/L in 15.7% and below 50 nmol/L in 47.9% of participants, with substantial variation according to geographical region, season, and population characteristics [9]. Although these data are not specific to infants, they illustrate the global magnitude of inadequate vitamin D status. Infancy represents a life stage of particular interest for vitamin D status, given the rapid growth and skeletal mineralization occurring during this period; severe and prolonged vitamin D deficiency at this stage may lead to hypocalcemia, impaired bone mineralization, and nutritional rickets [11,12].

Assessment of vitamin D status in infancy requires caution because the thresholds used to define deficiency, insufficiency, and adequacy are not universally standardized. Evidence suggests that thresholds derived largely from older populations may not be directly applicable to early infancy. In a study of healthy term infants, the inflection point of the relationship between serum 25(OH)D and parathyroid hormone (PTH) was estimated at 6.83 ng/mL, with the upper limit of the 95% confidence interval at 8.64 ng/mL, raising the possibility that some currently used thresholds may overestimate vitamin D deficiency in early infancy [13].

Serum 25(OH)D is the main biomarker used to assess vitamin D status because of its relatively long circulating half-life and its ability to reflect overall vitamin D supply and body stores [14,15]. Nevertheless, clinically relevant thresholds vary among organizations and clinical contexts. Concentrations around 20 ng/mL (50 nmol/L) are commonly considered adequate to prevent nutritional rickets, while lower concentrations indicate an increased risk of inadequate vitamin D status [11,16].

Vitamin D supplementation is an established component of preventive infant care. Evidence from systematic reviews and meta-analyses indicates that vitamin D supplementation increases circulating 25(OH)D concentrations in infants, with the magnitude of the response influenced by the administered dose and baseline vitamin D status [16,17,18]. In breastfed infants, the low concentration of vitamin D in human milk means that direct supplementation is generally required to ensure an adequate intake [19,20,21]. In formula-fed infants, vitamin D intake depends on the amount of fortified formula consumed and its vitamin D content; therefore, infants consuming insufficient amounts of fortified formula may require additional vitamin D supplementation [21,22,23].

Although 400 IU/day is widely recommended for healthy infants, the optimal dose in different clinical and nutritional circumstances remains an area of ongoing discussion [24,25,26]. A 2024 systematic review and network meta-analysis including 29 trials and 14 supplementation regimens found that daily doses of ≥400 IU increased serum 25(OH)D compared with no supplementation. However, doses ≥800 IU/day were associated with a higher risk of hypervitaminosis D and hypercalcemia, whereas 400–600 IU/day appeared to provide a favorable balance between efficacy and safety [18]. Adherence to supplementation recommendations is also variable, which may contribute to differences in vitamin D status among infants and young children [27].

Season is another potential determinant of vitamin D status because cutaneous synthesis of vitamin D depends on ultraviolet B radiation. At the population level, low 25(OH)D concentrations are generally more frequent during winter and early spring than during summer and autumn [9,28]. In infants, however, the contribution of season may be related to supplementation, feeding type, and sun exposure. Furthermore, in infants, dietary factors may be important determinants of vitamin D status even when seasonal variation is present [29].

In Romania, data on vitamin D status in children remain limited, particularly during infancy. A single-center study from Cluj-Napoca, in northwestern Romania, involving children aged 2–18 years reported vitamin D deficiency in 27% of participants and demonstrated significant differences according to age, sex, and season [30]. Additional evidence from a five-year retrospective analysis of children aged 0–18 years from Iași, in northeastern Romania, similarly documented substantial variability in serum 25(OH)D concentrations, with a mean of 27.4 ng/mL [31]. A large nationwide cross-sectional study of over 6600 individuals aged 0 to 85 years, based on samples collected across ten Romanian cities, similarly reported an increasing prevalence of insufficiency and deficiency with age; notably, infants aged 0–2 years had the highest prevalence of sufficient vitamin D status of all age groups, but potentially harmful concentrations (>100 ng/mL) were also disproportionately common in this age group, present in 17.3% of infants aged 0–1 year, an observation attributed in part to supplementation errors [32]. A retrospective national screening study conducted at a pediatric hospital in Timișoara, in western Romania, evaluated the effectiveness of Romania’s National Vitamin D Screening Programme in a population of children aged 0–18 years and reported an overall prevalence of vitamin D insufficiency of 23.9% among tested children, with age emerging as a consistent predictor of insufficiency [33]. Data from a Romanian perinatal cohort in northwestern Romania further indicate that vitamin D insufficiency and deficiency remain common in early life: among mothers and preterm infants born at ≤32 weeks of gestation, vitamin D deficiency was observed in 55.5% of mothers and 58.5% of infants, with severe deficiency (<10 ng/mL) present in approximately 20% of mothers and 16% of infants [34]. A study of pregnant women and their term neonates conducted in Târgu Mureș, in central Romania, emphasized the influence of maternal supplementation, diet, sun exposure, and socioeconomic background on neonatal vitamin D status [35]. Collectively, these findings provide relevant epidemiological context for the Romanian pediatric and perinatal population but cannot be directly extrapolated to infants aged 1–5 months, whose nutritional and metabolic characteristics differ substantially both from those of older children and from neonates, and who depend primarily on postnatal supplementation and feeding-related vitamin D intake. Considering these limitations, evaluating vitamin D status during early infancy and identifying the factors associated with serum 25(OH)D concentrations may help clarify the relationships between supplementation, feeding practices, and season in this particularly vulnerable period, for which data from Romania remain scarce. Accordingly, the present study aimed to assess vitamin D status in infants aged 1–5 months in northwestern Romania and to investigate its associations with vitamin D supplementation dose, feeding type, and season of blood sampling.

## 2. Materials and Methods

### 2.1. Participants and Data Collection

This was a retrospective, single-center, cross-sectional observational study. Data on vitamin D status were retrieved from the laboratory records of the Emergency Clinical Hospital for Children, Cluj-Napoca, a university-affiliated pediatric hospital located in northwestern Romania. Eligible participants were infants aged 1–5 months who were admitted to the hospital for specialized investigations between January and December 2023 and from whom a venous blood sample had been collected for determination of serum 25(OH)D concentration. The main reasons for admission were assessment of growth/nutritional status and scheduled investigations. Serum 25(OH)D was measured at the discretion of the attending physician, as part of the general assessment of the infant’s vitamin D status. Infants with prematurity (gestational age <37 weeks), chronic illness (e.g., genetic syndromes), or disorders of calcium/vitamin D metabolism were excluded from the study. Demographic and clinical information relevant to the study objectives, including age, sex, season of blood sampling, feeding type, and vitamin D supplementation dose, was extracted from the medical records for each participant. Feeding type and vitamin D supplementation dose were ascertained by parental report at the time of admission. Only the daily dose reported at admission was available; the duration of supplementation, its regularity, and adherence were not systematically recorded. Furthermore, the medical records did not document who had recommended the dose (e.g., the family physician or the pediatrician).

### 2.2. Sample Collection and Analysis

Vitamin D status was assessed by measuring serum 25-hydroxyvitamin D [25(OH)D]. Venous blood samples were collected as part of the routine admission blood work, at various times of the day and irrespective of feeding time, into sterile 4-mL vacutainer tubes without anticoagulant. Samples were centrifuged at 2000× *g* for 10 min to separate the serum. A volume of 40 µL of serum was required for the 25(OH)D assay.

Serum 25(OH)D concentrations were determined using a chemiluminescent immunoassay performed on the Mindray SAL 6000 analytical system (BS800 + CL 2000i; Shenzen Mindray Bio-Medical Electronics Co., Ltd., Shenzhen, China).

For the purposes of the present study, vitamin D status was categorized according to the following predefined thresholds: (i) vitamin D deficiency: <12 ng/mL; (ii) vitamin D insufficiency: 12 to <20 ng/mL; (iii) vitamin D sufficiency: 20–100 ng/mL; (iv) potentially excessive 25(OH)D concentration: >100 ng/mL. The term potentially excessive 25(OH)D concentration was used instead of vitamin D toxicity because an elevated serum 25(OH)D concentration alone does not establish clinical vitamin D intoxication, which generally requires evidence of disturbed calcium metabolism [24].

### 2.3. Statistical Analysis

Data were reported conform they type. Qualitative data were reported as absolute and relative frequencies. Quantitative data were first tested for distribution using the Shapiro-Wilk test. Mean and standard deviation were reported for data that proved to follow a normal distribution, and differences were tested with Student’s *t*-test (two groups) or ANOVA test (more than two groups). Median and IQR (interquartile range [Q1 to Q3], where Q represents the value of quartile) were reported for data without a normal distribution, and differences between groups were tested with non-parametric tests, the Kruskal-Wallis test for feeding type, vitamin D supplementation groups, and seasons. Post hoc analysis was conducted using Dwass-Steel-Critchlow-Fligner pairwise test when the Kruskal-Wallis test reached the significance threshold. Vitamin D values were also reported as minimum to maximum values. The distributions of vitamin D by groups were plotted following the method proposed by Weissgerber et al. [36] or box and whiskers. Statistical analysis was conducted with Jamovi software (v. 2.7.37.0). Statistical significance was set to 5%, *p*-values less than 0.05 showing statistically significant differences.

## 3. Results

We assessed vitamin D status in 219 infants, 124 girls (56.6%), and 95 boys (43.4%), respectively. The evaluated girls and boys had a similar median age (4 months), without statistically significant difference (*p*-value = 0.7562). The main characteristics of the evaluated cohort are presented in Table 1.

**Figure 1:**
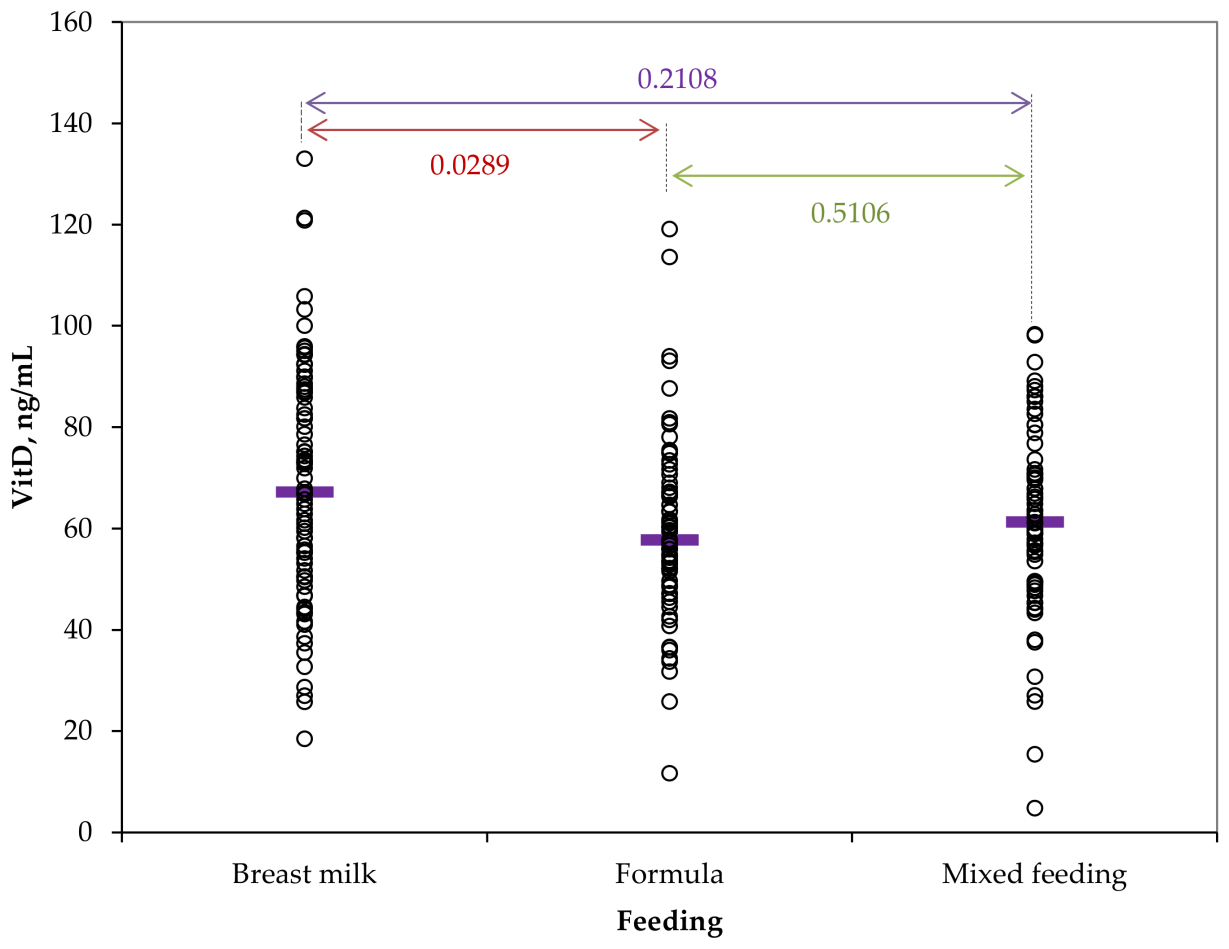
Distribution of serum concentrations of 25(OH)D (VitD, ng/mL) by type of feeding. The dots represent individual values; the line indicates the median.

The number of infants with different types of feeding was similar in the sample evaluated (Table 2, Figure 1), but Vitamin D supplementation was not homogeneous between different feeding groups (Table 2, Fisher’s exact test: *p*-value = 0.0134). The Kruskal-Wallis test identified statistically significant differences in vitamin D status between infants with different types of feeding (χ2(df) = 7.16 (2), *p*-value = 0.0279). The post hoc analysis demonstrated a statistically significant difference only between infants who were breastfed and those who were formula-fed (Figure 1).

Two infants with vitamin D deficiency were fed formula (one female infant) or mixed feeding (one male infant), while the two infants with vitamin D insufficiency were breastfed (one male infant) or mixed-fed (one female infant). Potentially excessive 25(OH)D concentrations were observed in eight infants (seven female and one male), six of whom were breastfed and two formula-fed (both female infants).

**Figure 2:**
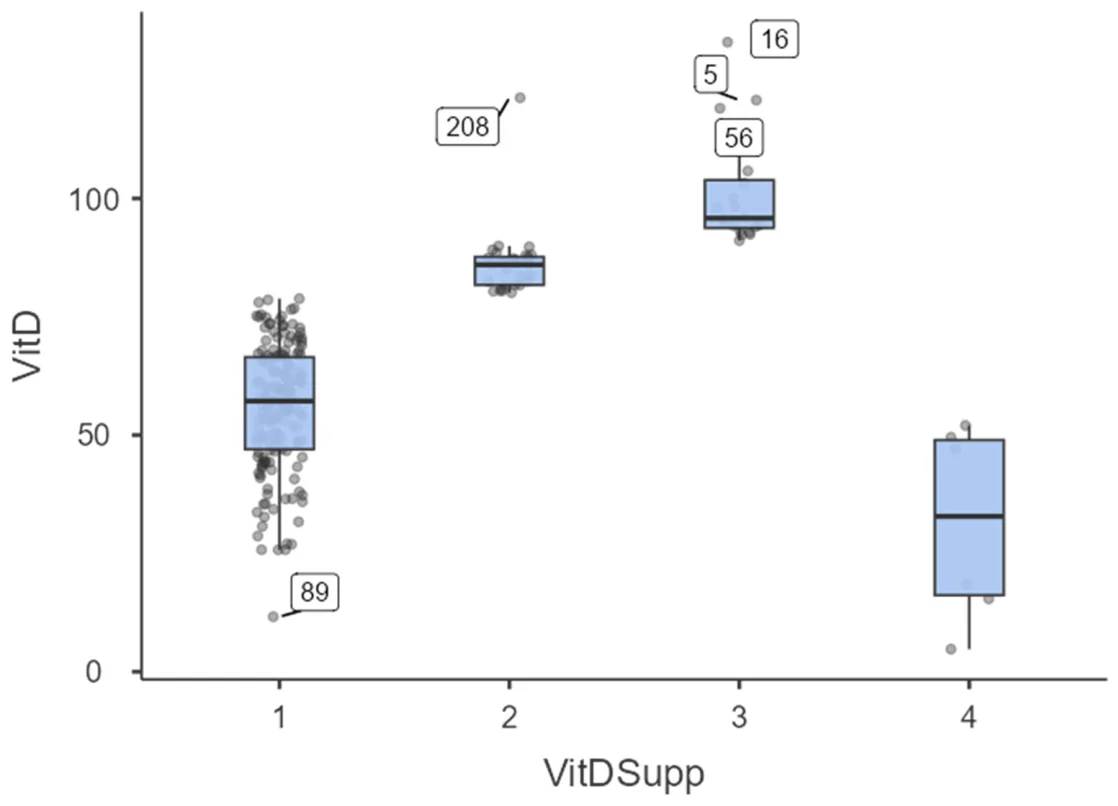
Distribution of serum concentrations of 25(OH)D (VitD, ng/mL) by vitamin D supplementation (VitDSupp). 1 =1000 IU/day; 2 = 1500 IU/day; 3 = 2000 IU/day; 4 = no supplementation. The dots represent individual values; the box is defined by the values of the first and third quartiles, with the line within the box indicating the median.

Most infants (74.9%) received a dose of 1000 IU/day vitamin D supplementation. As expected, serum 25(OH)D concentrations increased with the increase of vitamin D supplementation (Table 3, Figure 2), leading to statistically significant differences (Kruskal-Wallis test: χ2(df) = 120.1 (3), *p*-value < 0.0001). The post hoc analysis demonstrated a statistically significant difference between all pairs (Table 3).

Two infants with vitamin D deficiency received it intermittently 1000 IU/day or no supplementation, while the two infants with vitamin D insufficiency received no vitamin D supplementation. Potentially excessive 25(OH)D concentrations were observed in one infant who received 1500 IU/day and seven with 2000 IU/day.

**Figure 3:**
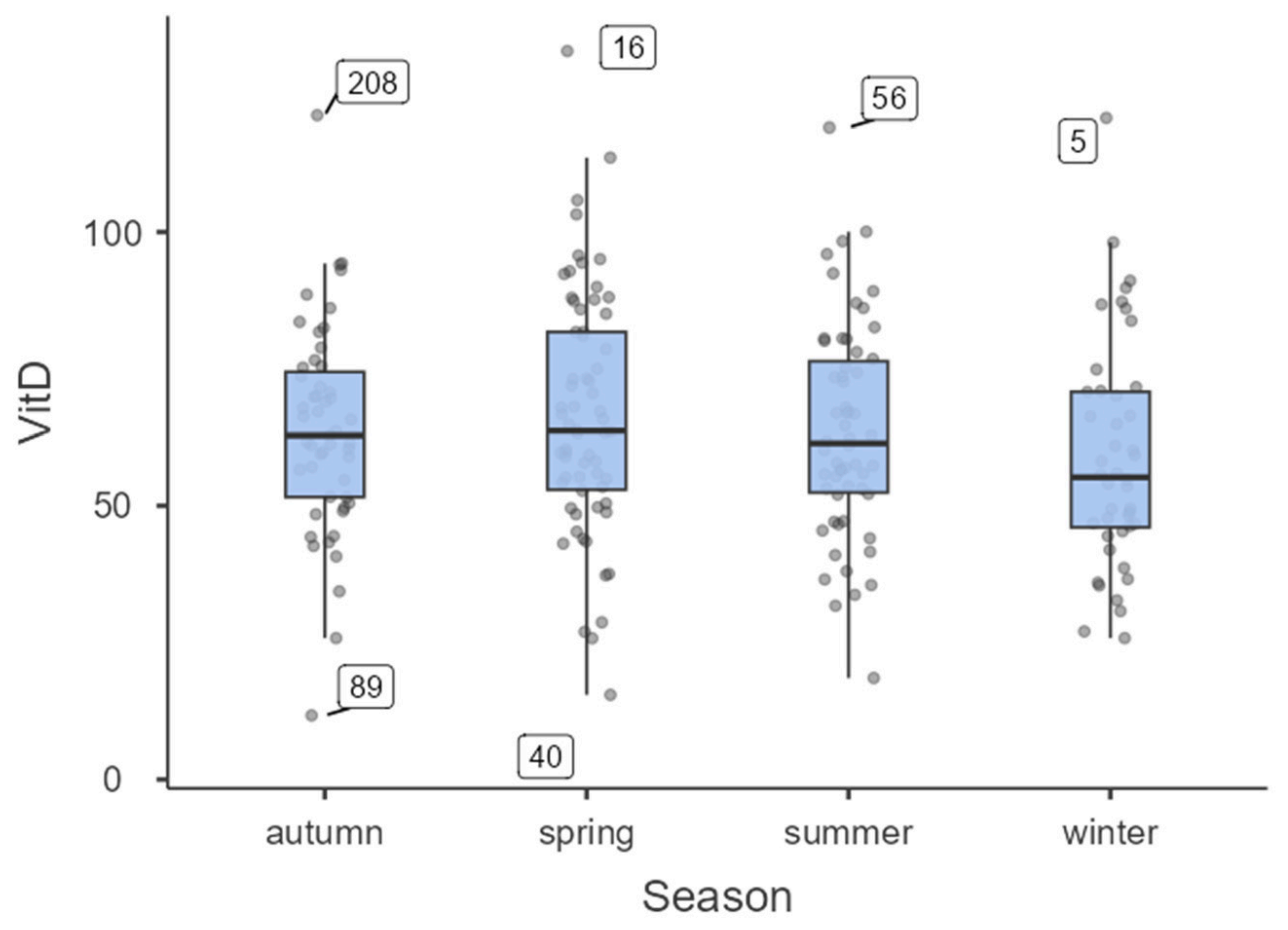
Distribution of serum concentrations of 25(OH)D (VitD, ng/mL) by season of blood sampling. The dots represent individual values; the box is defined by the values of the first and third quartiles, with the line within the box indicating the median.

The distribution of evaluated infants by season of blood sampling is uniform (Table 4). The serum concentrations of 25(OH)D had similar values and distribution (Table 4, Figure 3), without statistically significant differences between groups (ANOVA test: F(df1,df2) = 0.94 (3, 115.46), *p*-value = 0.4218).

## 4. Discussion

The median serum 25(OH)D concentration in our cohort was 61.24 [49.53–75.32] ng/mL, and 94.5% of infants had concentrations classified as sufficient according to the prespecified study thresholds. Vitamin D deficiency and insufficiency were each identified in 0.9% of participants, whereas 3.7% had 25(OH)D concentrations >100 ng/mL, which were classified as potentially excessive according to the study criteria. The main findings were higher serum 25(OH)D concentrations with higher reported supplementation doses and differences in serum 25(OH)D concentrations by feeding type. In contrast, no statistically significant seasonal variations in serum 25(OH)D concentrations were observed. Because the study was retrospective and hospital-based, and because supplementation doses were reported by parents without information on duration or adherence, these findings describe associations within this cohort and should not be interpreted as causal effects or extrapolated to the general infant population.

The low prevalence of vitamin D deficiency and insufficiency observed in our cohort should be interpreted cautiously, given the characteristics of the study population (i.e., a hospital-based sample from a single center) and the high prevalence of supplementation. Considerably higher prevalences of suboptimal vitamin D status have been reported in other populations of infants and young children. For example, a cross-sectional study conducted in Saudi Arabia, including 484 children aged ≤2 years, reported vitamin D deficiency in 24.6% and insufficiency in 45.9% of participants [37]. Similarly, a large retrospective study from Turkey, including 35,620 children aged 0–18 years, reported vitamin D deficiency in 22.2% and insufficiency in 31.2% of infants specifically [38].

Nevertheless, the interpretation of vitamin D status in early infancy is particularly challenging because the thresholds used to define deficiency and insufficiency are not universally standardized. In our study, the thresholds were prespecified as follows: deficiency, <12 ng/mL; insufficiency, 12–<20 ng/mL; sufficiency, 20–100 ng/mL; and potentially excessive concentrations, >100 ng/mL. These categories should not, however, be interpreted as universally established biological thresholds. Recent evidence suggests that the relationship between serum 25(OH)D and PTH in early infancy may be characterized by considerably lower concentration thresholds than some of those currently used in clinical practice. Yadav et al. identified an estimated 25(OH)D inflection point of 6.83 ng/mL, with an upper 95% confidence limit of 8.64 ng/mL, and suggested that currently used thresholds for vitamin D deficiency in early infancy may warrant reconsideration [13]. Lin (2025) similarly highlighted the need to reevaluate the applicability of conventional 25(OH)D cut-offs in early infancy [39]. Consequently, the very low prevalence of vitamin D deficiency observed in our cohort may reflect not only the effectiveness of supplementation but also the uncertainty surrounding the biological and clinical interpretation of vitamin D thresholds in young infants.

The predominance of an adequate vitamin D status observed in the present cohort is consistent with previous Romanian pediatric studies, which have generally reported an age-related gradient in vitamin D status, with suboptimal concentrations becoming more prevalent among older children and adolescents [31,32,33]. In particular, a large nationwide cross-sectional study found that infants aged 0–2 years had the highest prevalence of vitamin D sufficiency among all age groups, an observation attributed at least in part to the widespread use of vitamin D supplementation during infancy [32]. Indeed, there is increasing evidence demonstrating the efficacy of vitamin D supplementation in enhancing serum 25(OH)D concentrations [16,40]. Similarly, the high proportion of infants with sufficient vitamin D status in our study can be interpreted in the context of the high prevalence of supplementation in the cohort. Serum 25(OH)D concentrations were higher in each successive reported dose category, with medians of 32.8, 57.2, 86.0, and 95.8 ng/mL among infants receiving no supplementation, 1000, 1500, and 2000 IU/day, respectively. Differences between all supplementation groups were statistically significant. This dose-related pattern is consistent with previous intervention studies and meta-analyses [16,18]. In particular, the 2024 network meta-analysis by Abiramalatha et al., which included 29 trials evaluating 14 supplementation regimens, found that doses ≥ 400 IU/day increased mean 25(OH)D concentrations compared with no supplementation [18]. Nevertheless, several features of our data warrant caution. The doses were reported by parents and could not be verified, information on duration and adherence was not available, and the 1500 IU/day (*n* = 29), 2000 IU/day (*n* = 20), and unsupplemented (*n* = 6) groups were small. The magnitude of the differences between dose groups should therefore be regarded as approximate.

It is also important to emphasize that a higher serum 25(OH)D concentration should not automatically be interpreted as evidence of a proportionally greater clinical benefit. In a randomized clinical trial of infants with baseline 25(OH)D concentrations < 20 ng/mL, supplementation with 1000 IU/day resulted in higher serum 25(OH)D concentrations than 400 IU/day but did not provide an additional benefit in terms of bone mineral accretion [41]. These findings indicate that increasing circulating 25(OH)D beyond concentrations adequate for preventing deficiency does not necessarily translate into additional skeletal benefit. Although higher-dose supplementation has been investigated in selected infants at increased risk, such findings should not be extrapolated to routine prophylaxis in all healthy infants. Therefore, achieving the highest possible serum 25(OH)D concentration should not be considered a therapeutic goal in itself in the absence of a specific clinical indication.

In our study, 97.3% of infants received vitamin D supplements, and almost three-quarters received 1000 IU/day. This high uptake of vitamin D prophylaxis indicates broad awareness of the need for vitamin D supplementation in this age group, consistent with national guidance to initiate vitamin D supplementation from 7 days of life, including in preterm infants, and to continue the supplementation up to 12 months of age [42]. National guidance recommends a dose of 400–800 IU/day for eutrophic infants, a range broadly consistent with several international guidelines, which recommend a daily dose of 400 IU for all infants throughout their first year of life [24,25,26,42]. However, the doses recorded in our cohort were, in almost all cases, above this range, with even the most frequently administered dose (1000 IU/day) exceeding the upper recommended limit. This discrepancy likely reflects the prevailing clinical practice: informally, family physicians and pediatricians in this region may commonly recommend higher empirical doses. In addition, the oral cholecalciferol drops most commonly used in Romania deliver approximately 500 IU per drop [43]. When physicians aim for the upper end of the recommended range (800 IU/day), the smallest dose that reaches this target with such preparations is two drops, that is, 1000 IU/day; similarly, the doses of 1500 and 2000 IU/day correspond to three and four drops, respectively. Taken together, these findings suggest that, while awareness of the need for vitamin D prophylaxis appears to be high among caregivers and healthcare providers, the doses actually administered often exceed current national and international recommendations, underscoring the importance of aligning everyday prescribing and administration practices with evidence-based dosing guidance [24,25,26,42]. A network meta-analysis published in 2024 suggested that 400–600 IU vitamin D/day may provide a favorable balance between efficacy and safety, whereas doses ≥ 800 IU/day were associated with an increased risk of hypervitaminosis D and hypercalcemia in the analyzed trials [18]. Importantly, the latter findings should be interpreted in the context of the evidence base and should not be taken to mean that every infant receiving ≥800 IU/day will develop toxicity. In our cohort, eight infants (3.7%) had 25(OH)D concentrations >100 ng/mL; seven were reported to receive 2000 IU/day and one 1500 IU/day. Both doses exceed the Tolerable Upper Intake Level of 1000 IU/day established by the European Food Safety Authority for infants up to 6 months of age [44]. Given the small number of cases, the small size of the higher-dose groups, and the absence of data on calcium metabolism, this observation should be considered hypothesis-generating. The concentrations of 25(OH)D > 100 ng/mL in our study were classified as potentially excessive according to the prespecified criteria and were not equated with clinical vitamin D toxicity. Vitamin D toxicity is primarily a clinical and biochemical syndrome associated with excessive vitamin D exposure and dysregulated calcium homeostasis, particularly hypercalcemia and hypercalciuria. Serum 25(OH)D is an important biomarker of vitamin D exposure, but its concentration alone is insufficient to establish a diagnosis of vitamin D toxicity [45]. This distinction is particularly important for interpreting our findings because serum calcium, phosphate, PTH, and urinary calcium measurements were not available for infants with 25(OH)D concentrations > 100 ng/mL. Accordingly, within the present dataset, the term “potentially excessive 25(OH)D concentration” is more appropriate than “vitamin D toxicity”. This terminology avoids overinterpretation of an isolated biochemical measurement and acknowledges that clinical toxicity requires assessment of calcium homeostasis and the broader clinical context.

The presence of elevated 25(OH)D concentrations among infants receiving higher supplementation doses also highlights the importance of preventing dosing errors. Case reports have described severe hypercalcemia in infants following accidental administration of excessively concentrated vitamin D preparations or errors in dose measurement [46,47]. Prolonged exposure to excessive vitamin D may also result in persistent biochemical abnormalities after supplementation has been discontinued [48]. These observations emphasize the importance of providing parents and caregivers with clear instructions regarding the concentration of the preparation, the prescribed dose, the volume to be administered, and the avoidance of concomitant use of multiple vitamin D-containing products.

Another important finding of our study was the significantly higher serum 25(OH)D concentration among breastfed infants compared with formula-fed infants. However, this association should be interpreted cautiously. Human milk naturally contains relatively low concentrations of vitamin D (< 50 IU/L) and therefore cannot, by itself, explain the higher 25(OH)D concentrations observed in breastfed infants, whereas infant formula is fortified with vitamin D [21]. Moreover, the distribution of supplementation doses differed significantly between feeding groups (Table 2): 34.2% of breastfed infants received 1500–2000 IU/day, compared with 11.4% of formula-fed and 20.3% of mixed-fed infants, and 13 of the 20 infants receiving 2000 IU/day were breastfed. This difference in supplementation is likely to contribute to the higher 25(OH)D concentrations observed in breastfed infants; however, because vitamin D intake from fortified formula could not be quantified, differences in adherence, the accuracy of reported doses, or other unmeasured factors may also play a role. Systematic reviews indicate that direct vitamin D supplementation of breastfed infants increases serum 25(OH)D concentrations and reduces the risk of inadequate vitamin D status [19,20]. Maternal vitamin D supplementation during lactation may also influence infant vitamin D status [49,50]. Thus, feeding type should be regarded primarily as a marker of a broader nutritional and behavioral context rather than interpreted as an independent causal determinant of serum 25(OH)D concentrations. Evidence from systematic reviews also indicates that maternal postpartum supplementation and intermittent infant supplementation have been investigated as alternatives to daily infant supplementation, although the evidence remains insufficient to support a change in routine policy [20].

Observational studies have further examined the determinants of vitamin D status in infants. A study of Chinese infants evaluated at 6 months identified season, vitamin D supplementation, and feeding patterns among factors associated with serum 25(OH)D concentrations [51]. Similarly, a study of Japanese infants aged 1–2 months found that formula intake was strongly associated with vitamin D status, while seasonal variation in 25(OH)D concentrations became apparent from approximately 2 months of age, particularly among breastfed infants [52]. Together, these findings highlight the need to consider feeding practices, supplementation, and seasonal factors when interpreting vitamin D status during early infancy.

Regarding seasonal variation specifically, although cutaneous vitamin D synthesis is dependent on exposure to ultraviolet B radiation, we did not identify statistically significant seasonal differences in serum 25(OH)D concentrations in our cohort. Median concentrations ranged from 55.2 ng/mL in winter to 63.7 ng/mL in spring, but the overall seasonal difference was not statistically significant. The absence of statistically significant seasonality may be explained, at least in part, by the high prevalence of vitamin D supplementation in our cohort, which may have attenuated the contribution of seasonal variation in ultraviolet exposure to circulating 25(OH)D concentrations. During early infancy, the influence of season may differ according to feeding type. In a Japanese cohort of 692 infants aged 1–2 months, seasonal variation in 25(OH)D concentrations became apparent at 2 months, particularly among breastfed infants, whereas formula intake was a major determinant of vitamin D status [52]. Thus, the absence of a statistically significant seasonal association in our study does not exclude a biological effect of season; rather, it suggests that, under conditions of widespread supplementation, the contribution of season may be smaller than that of exogenous vitamin D intake.

Finally, the clinical implications of our findings should be considered carefully, because our research was not designed to define an optimal dose or to assess clinical outcomes. Our findings show that, in this setting, supplementation was nearly universal, the reported doses frequently exceeded national and international recommendations, and concentrations > 100 ng/mL were confined to infants reported to receive the highest doses. Current pediatric recommendations generally support 400 IU/day for all infants [11,23,24,25,26]. Our observations therefore support reinforcing adherence to recommended prophylactic doses rather than the routine use of higher doses in healthy infants. Higher doses are appropriate for the treatment of confirmed deficiency under medical supervision, because even therapeutic regimens may cause asymptomatic hypercalcemia [53].

Overall, our findings support the importance of maintaining adequate vitamin D status while avoiding unnecessary exposure to high supplementation doses. The high proportion of infants with sufficient 25(OH)D concentrations in our cohort is consistent with the effectiveness of widespread supplementation in this population. At the same time, the identification of potentially excessive 25(OH)D concentrations among infants receiving higher supplementation doses highlights the need to balance prophylactic efficacy with safety. The objective of supplementation should therefore not be to achieve the highest possible serum 25(OH)D concentration, but rather to maintain an adequate vitamin D status using doses appropriate to the infant’s age, feeding pattern, and individual clinical circumstances.

The findings of the present study should be interpreted in light of several strengths and limitations. The main strengths of this study include the relatively large sample size for a single-center study and the availability of information on three clinically relevant factors potentially associated with vitamin D status: supplementation dose, feeding type, and season of blood sampling. In addition, the study provides data specifically on early infancy, an age group for which Romanian data on serum 25(OH)D concentrations remain limited. Notwithstanding these strengths, the study also has several limitations that warrant consideration. First, the observational nature of the study precludes the establishment of causal relationships between vitamin D supplementation, feeding type, season, and serum 25(OH)D concentrations. Second, information regarding adherence to supplementation, duration of supplementation, maternal vitamin D status, the precise contribution of vitamin D from infant formula, and individual ultraviolet radiation exposure was not comprehensively available. These factors may have contributed to residual confounding and may partly explain some of the observed associations. Supplementation doses were reported by parents at admission and could not be verified against prescriptions. Because the volume of formula consumed was not recorded, vitamin D intake from formula could not be quantified; infant formula marketed in the European Union contains 2–2.5 µg (80–100 IU) of vitamin D per 100 kcal, or approximately 500–700 IU per liter [54], so the total vitamin D intake of formula-fed and mixed-fed infants was probably higher than the supplement dose alone indicates. Furthermore, owing to the retrospective design, specific baseline characteristics of the infants, such as body weight, length, birth weight, skin pigmentation, and ethnicity, were not included in the study dataset, although these factors may influence vitamin D status. Third, the number of infants in certain subgroups, particularly those who did not receive supplementation and those with low vitamin D status, was relatively small, limiting the statistical power and precision of subgroup comparisons. Fourth, participants were recruited from infants admitted to a tertiary pediatric hospital for specialized investigations rather than from a general population or a routine well-child screening program. Although infants with prematurity, chronic illness, and disorders of calcium/vitamin D metabolism were excluded, the underlying reasons for hospital admission were not restricted to conditions related to vitamin D status. This hospital-based, clinically referred sample may therefore not be representative of the broader population of healthy infants in this age group, and the possibility of selection bias related to the indication for admission cannot be excluded. Another important limitation concerns the interpretation of vitamin D status categories. Thresholds used to define vitamin D deficiency and insufficiency in early infancy are not universally standardized, and the biological relevance of currently used cut-offs remains under discussion [13,39]. Accordingly, the prevalence estimates reported in the present study may vary depending on the thresholds applied. In addition, the analytical method used to determine serum 25(OH)D concentrations may influence the measured values and, consequently, the classification of individual participants. Finally, the eight infants classified as having potentially excessive 25(OH)D concentrations could not be evaluated for clinical or biochemical vitamin D toxicity because measurements of serum calcium, phosphate, PTH, and urinary calcium excretion were not available. Therefore, these findings should be interpreted as evidence of elevated biochemical exposure to vitamin D rather than as confirmed vitamin D intoxication. 

## 5. Conclusions

In this single-center, hospital-based study, most infants aged 1–5 months in northwestern Romania had serum 25(OH)D concentrations in the sufficient range, in a context of near-universal vitamin D supplementation. Higher reported supplementation doses were associated with higher 25(OH)D concentrations, whereas no statistically significant seasonal variation was observed. Concentrations > 100 ng/mL were found in a small number of infants, almost all reported to receive 2000 IU/day. In the absence of data on calcium metabolism, these cannot be interpreted as vitamin D toxicity, but they support adherence to recommended prophylactic doses. Given the retrospective design and parent-reported supplementation data, prospective, multicenter studies with verified dosing and assessment of calcium metabolism are needed to confirm these findings and to guide supplementation practices in this population.

## Figures and Tables

**Table 1:** Characteristics of the evaluated cohort of infants.

Variable	Value
Age (months) ^a^	4 [3 to 5]
Season of blood sampling ^b^	
Autumn	51 (23.3)
Spring	66 (30.1)
Summer	58 (26.5)
Winter	44 (20.1)
Feeding mode ^b,c^	
Breast milk	79 (36.1)
Formula	70 (32.0)
Mixed feeding	69 (31.5)
Vitamin D supplementation ^b^	
1000 IU/day	164 (74.9)
1500 IU/day	29 (13.2)
2000 IU/day	20 (9.1)
No supplementation	6 (2.7)
Vitamin D status ^d^	
Deficient	2 (0.9)
Insufficient	2 (0.9)
Sufficient	207 (94.5)
Potentially excessive	8 (3.7)
25(OH)D serum values (ng/mL) ^a^	61.24 [49.53 to 75.32]

^a^ Median [IQR]; ^b^ number (%); ^c^ one missing data; ^d^ Vitamin D status was assessed based on serum 25(OH)D concentration, categorized as deficient (<12 ng/mL), insufficient (12 to <20 ng/mL), sufficient (20–100 ng/mL), or potentially excessive (>100 ng/mL).

**Table 2:** 25(OH)D Vitamin D supplementation doses and 25(OH)D serum concentrations (ng/mL) by type of feeding.

	Breast Milk (*n* = 79)	Formula (*n* = 70)	Mixed Feeding (*n* = 69)
Vitamin D supplementation			
1000 IU/day 1500 IU/day 2000 IU/day no supplementation	51 (64.6) 14 (17.7) 13 (16.5) 1 (1.3)	60 (85.7) 4 (5.7) 4 (5.7) 2 (2.9)	52 (75.4) 11 (15.9) 3 (4.3) 3 (4.3)
25(OH)D serum concentrations (ng/mL)			
Median [Q1 to Q3] {minimum to maximum}	67.1 [51.1 to 86.9] {18.5 to 133}	57.7 [49 to 67.8] {11.6 to 119.1}	61.2 [49.4 to 71.6] {4.8 to 98.3}

**Table 3:** 25(OH)D serum concentrations (ng/mL) by pattern of vitamin D supplementation.

	1000 IU/day (*n* = 164)	1500 IU/day (*n* = 29)	2000 IU/day (*n* = 20)	No Supplementation (*n* = 6)
Median [Q1 to Q3]	57.2 [47 to 66.5]	86 [81.7 to 87.7]	95.8 [93.8 to 103.9]	32.8 [16.2 to 49]
{minimum to maximum}	{11.6 to 78.8}	{80.1 to 121.3}	{91.1 to 133}	{4.8 to 52}
Matrix of *p*-values: Dwass-Steel-Critchlow-Fligner pairwise test				
1000 IU/day		<0.0001	<0.0001	0.0213
1500 IU/day			<0.0001	0.0008
2000 IU/day				0.0015

**Table 4:** 25(OH)D serum concentrations (ng/mL) by season of blood sampling.

	Autumn (*n* = 51)	Spring (*n* = 66)	Summer (*n* = 58)	Winter (*n* = 44)
Median [Q1 to Q3]	62.8 [51.6 to 74.4]	63.7 [52.9 to 81.7]	61.4 [52.4 to 76.4]	55.2 [46.1 to 70.8]
{minimum to maximum}	{11.6 to 121.3}	{4.8 to 133}	{18.5 to 119.1}	{25.8 to 120.8}

## Data Availability

The data presented in this study are available on request from the corresponding author.
